# Morphometric evaluation of seminiferous tubule and proportionate numerical analysis of Sertoli and spermatogenic cells indicate differences between crossbred and purebred bulls

**DOI:** 10.14202/vetworld.2015.645-650

**Published:** 2015-05-22

**Authors:** Utkarsh K. Tripathi, Shivani Chhillar, A. Kumaresan, M. K. Muhammad Aslam, S. K. Rajak, Samiksha Nayak, A. Manimaran, T. K. Mohanty, Savita Yadav

**Affiliations:** 1 Theriogenology Laboratory, Animal Reproduction, Gynaecology & Obstetrics, National Dairy Research Institute, Karnal, Haryana, India; 2 Artificial Breeding Research Centre, National Dairy Research Institute, Karnal, Haryana, India; 3 Department of Biophysics, All India Institute of Medical Sciences, New Delhi, India

**Keywords:** crossbred bulls, purebred bulls, seminiferous tubules, Sertoli cells, testicular histology

## Abstract

**Aim:**

The present study compared the testicular cytology and histology between crossbred (Holstein–Friesian [HF] × Tharparkar) and purebred (HF and Tharparkar) bulls to find out differences if any.

**Materials and Methods:**

Four peripubertal bulls from each breed were utilized for the study. Through percutaneous needle aspiration biopsy, Sertoli and spermatogenic cells were extracted, and morphometry was studied. For histological studies, testicular tissues obtained through unilateral castration were utilized. Sertoli cells specific GATA4 antibody was used to study the population of Sertoli cells in the seminiferous tubule through immunofluorescence.

**Results:**

The testicular weight, volume, and scrotal circumference differed significantly among the breeds. The diameter and area of the seminiferous tubule was high in HF, followed by Karan Fries (KF), and Tharparkar bulls. However, the degree of compactness, based on qualitative evaluation, was high in Tharparkar followed by KF and HF bulls. The intensity of Leydig cells was higher in Tharparkar bulls followed by KF and HF. The proportion of Sertoli cells was higher (p<0.05) in HF and Tharparkar bulls compared to KF bulls.

**Conclusion:**

It may be concluded that variations exist in testicular components of the breeds studied and the proportion of Sertoli cells in relation to spermatogenic cells was significantly lower in crossbred bulls compared to purebred bulls.

## Introduction

Crossbreeding of *Bos indicus* with genetically superior exotic breeds of *Bos taurus* such as Holstein Friesian (HF) is being practiced in developing countries to improve the milk productivity. However, the males produced through crossing of *B. taurus* with *B. indicus* suffer from serious infertility/sub-fertility problems [1,2]. More than 50% of crossbred young bulls, which are progenies of elite dams and proven sires, inducted for semen collection are rejected for further use because of poor semen quality and low cryosurvivability of spermatozoa [1,3,4]. In crossbred bulls, we observed that the ejaculate rejection rate (owing to poor initial semen qualities) ranged from 23.02% to 100%, with the average of 52.46% [2]. There are also reports citing that the sperm functions and seminal plasma composition are altered in crossbred bulls [5,6]. On the other hand, the existing literature suggests that poor semen quality is not a major problem in exotic or indigenous bulls [3] indicating a possible effect of crossbreeding on sperm production or spermatogenesis process. The reason for production of low quality semen in crossbred bulls, even during the best breeding season, has not been identified yet.

It is well known that production of fertile spermatozoa is a consequence of normal mitosis and meiosis of germ cell and proper function of both the germ cells and Sertoli cells [7]. The number of germ cells supported by a single Sertoli cell is the best reflection of the functional efficiency of this cell and is usually highly correlated with spermatogenic efficiency [8]. It is also reported that the daily spermatozoa production can be accurately obtained from the total number of Sertoli cells per testis and the number of spermatids per Sertoli cell [9]. Recently, we reported the proteomic differences in Sertoli and spermatogenic cells between purebred and crossbred pubertal bulls [10]. Presently, we do not have any published information on morphology/biometry of seminiferous tubule, spermatogenic cells and Sertoli cells in crossbred bulls and how it differ from purebred bulls. Understanding these basic features would help us in improving our knowledge on spermatogenic process in these species.

In this context, the present investigation was undertaken to study the morphological and biometrical characteristics of seminiferous tubules, spermatogenic and Sertoli cells in purebred (indigenous and exotic), and crossbred peripubertal bulls.

## Materials and Methods

### Ethical approval

The experimental procedures were approved by the Institutional Animal Ethics Committee. The present investigation was carried out at the Livestock Research Centre, National Dairy Research Institute (NDRI), Karnal, India.

### Experimental animals and their management

A total of 12 peripubertal bulls (age 10 months), four each from HF (exotic purebred), Karan Fries (KF) (HF × Tharparkar crossbred; 50-75% HF inheritance), and Tharparkar (Indigenous purebred), maintained at Livestock Research Centre were utilized for the study. Up to 6 months of age, the calves were reared in groups and fed as per NRC standards. After 6 months of age, calves were transferred to semi loose housing system and fed with compound feed having soybean cake, wheat bran, rice bran, and *ad libitum* green fodder and clean drinking water. Concentrate mixture was fed to the bulls @1.25 kg/animal/day.

### Percutaneous needle aspiration cytology of testis and estimation of cell counts

To study the morphology and biometry of individual testicular cells, needle aspiration biopsy was carried out as per the standardized procedure [11]. Two aspirations were obtained from each of the experimental animals. The aspirate was expelled on a clean glass slide, a thin smear was prepared and fixed in methanol for 15 min, washed in running tap water for 1 min, air dried, and then stained with diluted May-Grunwald stain (1:1 with phosphate- buffered saline [PBS]) for 15 min. The smear was washed with tap water and air dried. Diluted Giemsa stain (1:3 with PBS) was then poured on the smear and allowed to stain for 45 min before washing and air drying. The smear was examined at ×400 and ×1000 magnifications (SMZ 100/SMZ 800, Nikon, Tokyo, Japan) and measurements were recorded using the pre-calibrated software (NIS-Elements, Basic Research, Tokyo, Japan). Total 200 clearly identifiable cells were counted from each smear for analyzing the cell number and morphometry of various testicular cells.

Different spermatogenic cells (spermatogonia, spermatocytes, and spermatids) and Sertoli cells were identified according to their distinct morphological characteristics as described by [12] and [13] and counted. The diameter and area of spermatogonia, spermatocytes and spermatids, and Sertoli cells were determined using pre-calibrated measuring eyepiece of phase contrast microscope and software.

### Unilateral castration of peripubertal bulls

Unilateral castration was performed as per the standard procedure and under strict aseptic conditions by veterinarians. Briefly, prior to castration the bulls were sedated with xylazine hydrochloride (Xylaxin, Indian Immunologicals, Hyderabad, India) at the dosage rate of 0.25 mL/50 kg body weight. The site of surgery was shaved and cleaned thoroughly with antiseptics. After local infiltration of 5-8 mL of 2% lignocaine (Cadila Healthcare Ltd., Ahmedabad, India) at the level of the spermatic cord, an incision was given at lower part of scrotum with the help of a BP blade (No. 23) with knife. One of the testes was exposed and the related spermatic cord was ligated tightly using catgut. After ligation, intact testis was removed and placed in individual sterile containers containing normal saline with penicillin streptomycin. Due post-operative care was given to the animals after castration as per the standard veterinary practice.

### Histological examination of testicular tissue

Testicular tissue fixed in Bouin’s solution (Qualichems, Vadodhara, India), were dehydrated in various grades of ethanol, cleared in benzene, and finally embedded in paraffin wax blocks. Sections (5-7 µm thick) were prepared using rotary microtome and mounted on albumenized glass slides. For studying the histoarchitecture of testicular tissue, the sections were stained using standard protocol of hematoxylin/eosin stain [14]. Stretched sections of testicular tissues (all the three groups simultaneously) were dewaxed in xylene (Xylene GR, Merck, Darmstadt, Germany) for 15-20 h. Stretched sections on the slides were downgraded through various grades of ethanol (Pro analysis ethanol, Merck, Darmstadt, Germany) to water and then stained with hematoxylin for 30 min. After up gradation up to 90% ethyl alcohol for dehydrating the slides were stained in 1% alcoholic eosin for 30 s followed by final dehydration in two changes of absolute ethanol for 10 min. Then the slides were transferred for cleaning to xylene and mounted in DPX (18404, DPX Mountant, Qualigens, Fisher Scientific, Mumbai, India). The nuclei were observed blue and the cytoplasm pink after the staining.

### Morphometric evaluation of seminiferous tubules

From each section, seminiferous tubule diameter and area (essentially from circular tubular cross sections) was determined using pre-calibrated measuring eyepiece. The sections were examined at × 400 and ×1000 magnifications (SMZ 100/SMZ 800, Nikon, Tokyo, Japan) and measurements were made using the software (NIS-Elements, Basic Research, Tokyo, Japan). About 20 sections of seminiferous tubules that were round or nearly round were chosen randomly and measured for each group. The tubular diameter was measured at ×400 magnification. The diameter of the seminiferous tubule was measured across the minor and major axes and the mean diameter was obtained.

### Immunohistochemistry for localization of sertoli cells and leydig cells

Immunohistochemistry was carried out as per the procedure given by [15]. Briefly, stretched tissue sections were dewaxed in xylene and rehydration was done in various grades of ethanol for 15-20 h at room temperature (22-25°C) and immersed in absolute alcohol (100%) for 15 min. Rehydration was done in various grades of ethanol (90%, 70%, 50%, and 30%) and finally in de- ionized water. For antigen retrieval, slides were immersed in 1 mM ethylenediaminetetraacetic acid (EDTA) (pH 8) buffer and were given heat treatment at 800 W for 5 min in a microwave oven at 100%. The same treatment was repeated at 20% for 5 min and the slides were cooled for 1 h in EDTA buffer and washed in de-ionized water and 0.9% PBS. For blocking non-specific staining sites, sections were treated with 10% BSA. Sections were then washed in PBS twice for 5 min each. Then the slides were treated with fluorescein isothiocyanate conjugated GATA 4 antibody (orb 13985, biorbyt, Riverside, UK). The slides were then incubated in a humidified chamber at 37°C for 4 h and then washed in PBS twice, for 5 min each. Slides were mounted using anti-fading mounting media containing 1, 4-Diazobicyclo-2, 2, 2-octane. Smears were examined under fluorescent microscope using ×40 and ×100 objectives (at wavelength 562 nm).

### Statistical analysis

Descriptive statistics was calculated for the tubular dimensions and the results are expressed as mean ± standard error (SE). One-way ANOVA was performed to compare the parameters in different breeds of bulls. When the p<0.05, the means were considered to be significantly different among the groups. The analyses were performed using Sigma Plot 11^®^ Software Package (Systat software Inc., Chicago, USA).

## Results

The testicular weight, width, volume, and scrotal circumference in Holstein bulls was higher (p<0.05) compared to either KF or Tharparkar bulls (Table-1).

**Table-1:** Testicular morphological parameters (Mean±SE) in different breeds of peripubertal bulls.

Parameter^A^	HF	KF	Tharparkar
Testicular weight (g)	53.8±3.5^c^	39.4±2.4^a^	14.5±1.2^b^
Testicular length (cm)	9.5±0.2^a^	8.5±0.6^a^	5.8±0.3^b^
Testicular width (cm)	4.8±0.3^c^	3.5±0.2^a^	2.6±0.1^a^
Testicular volume (cm^3^)	115.3±20.4^c^	55.6±9.4^a^	21.1±2.4^b^
Scrotal circumference (cm)	28.7±0.5^c^	23.6±0.6^a^	20.2±0.2^b^

A Length and width of each testis were measured with calipers. Testicular volume was calculated using a formula (volume=0.5236×length×width^2^), HF=Holstein Friesian, KF=Karan Fries. Means with different superscripts within the same row differ significantly (p<0.05), SE=Standard error

### Seminiferous tubules

The seminiferous tubules appeared as tortuous, round and oblong in outline, varying in appearance due to the complex coiling of the tubules at different angles and levels. The diameter of the seminiferous tubule ranged from 140 µm to 351.5 µm in HF bull calves, from 137 µm to 265 µm in KF bull calves and from 63 µm to 145 µm in Tharparkar bull calves, respectively. The mean (SE) diameter of the seminiferous tubule in HF, KF, and Tharparkar bull was 228.04±8.94, 183.43±4.57, and 111.71±2.53 µm, respectively (Table-2) and the differences were significant (p<0.001).

**Table-2:** Diameter and area (mean±SE) of seminiferous tubules in peripubertal bulls.

Parameter	Breed		

	HF	KF	Tharparkar
Diameter (µm)	228.04±8.94^c^	183.43±4.57^b^	111.71±2.53^a^
Area (µm^2^)	43309.73±3338.24^c^	27145.74±1454.68^b^	10021.93±436.03^a^

Means with different superscripts within the same row differ significantly (p<0.001), SE=Standard error, HF=Holstein Friesian, KF=Karan Fries

**Figure-1:**
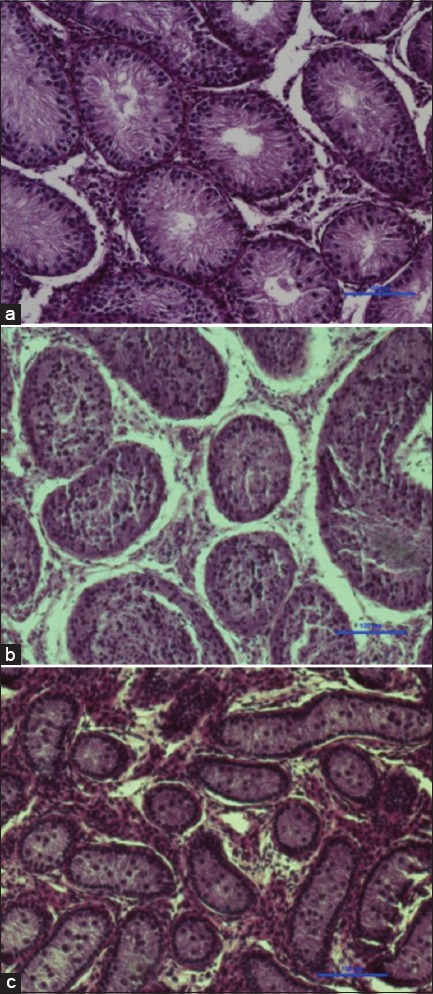
Testicular section indicating the compactness of seminiferous tubule, intensity of Leydig cells, and status of spermatogenesis in peripubertal bulls (×400); (a) Holstein Friesian, (b) Karan Fries, (c) Tharparkar.

The area of seminiferous tubule was higher in HF bulls compared to either KF or Tharparkar bulls. The area of the seminiferous tubule ranged from 15400 µm^5^ to 97096 µm^2^ with a mean of 43309.73±3338.24 µm^2^ in HF bulls. In KF bulls, the diameter of seminiferous tubules ranged from 14747 µm^2^ to 57172 µm^2^ with a mean of 27145.74±1454.68 µm^2^. However, in Tharparkar bulls, the area of the seminiferous tubule ranged from 3094 µm^2^ to 16577 µm^2^ only with a mean (±SE) of 10021.93±436.03 µm^2^. The degree of compactness, based on qualitative evaluation, was high in Tharparkar followed by KF and HF bulls (Figure-1). Similarly, the intensity of Leydig cells was higher in Tharparkar bulls followed by KF and HF bulls.

### Spermatogenic cells

Spermatogonia were observed in all the breeds however, their distribution in the seminiferous tubules differed among breeds. The spermatogonial cells were tightly packed in the basal compartment of seminiferous tubule in Tharparkar bulls compared to either HF or KF bulls (Figure-1). The arrangement of spermatogonia was very systematic and concentric in Tharparkar bulls. The proportion of spermatogonia to the total cells (spermatogenic and Sertoli cells) in the seminiferous tubule was 12.83±1.95 in KF, 11.16±1.42 in HF, and 12.16±1.16 in Tharparkar bulls (Table-3). The diameter and cell area of spermatogonial cells in HF, KF, and Tharparkar bulls are given in Table-4. The diameter of spermatogonia was significantly higher in HF and KF bulls compared to Tharparkar bulls, which was reflected in the cell area also. The diameter and cell area of spermatocytes in HF and KF bulls are given in Table-4. The diameter and cell area of spermatocytes in HF bulls did not differ with KF bulls, but differed significantly from Tharparkar bulls. As in case of spermatogonial cells, the proportion of spermatocytes also did not differ significantly among the breeds.

**Table-3:** Proportionate numerical analysis of different testicular cells (mean±SE) in peripubertal bulls.

Breed	Cells (%)		

	Spermatogonia	Spermatocytes	Sertoli cells
HF	11.16±1.42	14.66±2.98	11.62±0.80^a^
KF	12.83±1.95	13.16±1.88	8.5±0.67^b^
Tharparkar	12.16±1.16	11.16±1.22	14.66±2.71^a^

Means with different superscripts within the same row differ significantly (p<0.001), SE=Standard error, HF=Holstein Friesian, KF=Karan Fries

**Table-4:** Diameter and area (mean±SE) of cells of seminiferous tubules in peripubertal bulls.

Parameter	Breed		

	HF	KF	Tharparkar
Spermatogonia diameter (µm)	9.81±0.65^b^	9.26±0.39^b^	7.47±0.28^a^
Spermatogonia cell area (µm^2^)	74.59±2.26	69.29±4.38^b^	44.99±3.38^a^
Spermatocyte diameter (µm)	9.21±0.68	8.93±0.28	6.72±0.31
Spermatocyte area (µm^2^)	67.01±9.84	63.40±3.24	35.55±3.27
Sertoli cell diameter (µm)	15.98±0.62	15.55±0.82	14.26±0.55
Sertoli cell area (µm^2^)	208.32^a^±11.23	185.59±17.46	159.86^b^±10.96

Means with different superscripts within the same row differ significantly (p<0.001), SE=Standard error, HF=Holstein Friesian, KF=Karan Fries

### Sertoli cells

**Figure-2:**
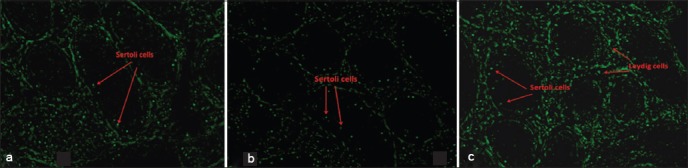
Immunolocalization of Sertoli cells using GATA4 antibody in peripubertal bulls; (a) Holstein Friesian, (b) Karan Fries, (c) Tharparkar.

Although smaller in size, the diameter of Sertoli cells of Tharparkar bulls (14.26±0.55) did not differ significantly from either KF (15.55±0.82) or HF (15.98±0.62) bulls. However, the area of Sertoli cells was significantly (p<0.05) higher in HF bulls compared to Tharparkar bulls. Unlike in case of spermatogonia and spermatocytes, the number of Sertoli cells differed significantly (p<0.05) among breeds (Table-3). In HF bulls, the proportion of Sertoli cells to the total cells (spermatogenic and Sertoli cells) was 11.62±0.8, while it was 14.66±2.71 in Tharparkar bulls. The proportion of Sertoli cells in KF bulls was only 8.5±0.67, which was significantly (p<0.05) lower compared to both HF and Tharparkar bulls. The mean (±SE) GATA4 positive cells in seminiferous tubules of HF, KF, and Tharparkar breeds were 14.7±0.9, 6±0.6 and 13±0.6, respectively (Figure-2).

## Discussion

Sub-fertility/infertility is very high in crossbred bulls compared to either indigenous or exotic bulls [1,4]. Crossbred bulls produce more numbers of ejaculates with poor spermatozoa quality [16]. Spermatozoa are the finished product from seminiferous tubule. Since the quality of ejaculated spermatozoa reflects the quality of precursor cells, we studied the morphology and biometry of the seminiferous tubule and its component cells in crossbred bulls and compared with purebred and indigenous bulls.

The diameter of the seminiferous tubule was high in HF bulls, followed by KF and Tharparkar. The diameter of seminiferous tubule observed in the present study is in agreement with those reported earlier [17-19]. It has been reported that the testicular size is bigger in exotic breeds compared to indigenous breeds [16,20]. Since the components/compartments of the testis contribute to its size and volume, it is expected that the seminiferous tubules are also bigger in breeds with big testis, which is supported by the results of our study. The area of seminiferous tubule differed significantly among breeds, which might be attributed to breed difference. Smaller testicular volumes, together with minor values of tubular diameters might indicate a decrease in spermatogenesis [18]. The increase in spermatogenesis process leads to increase the diameter and thickness of seminiferous tubules and our findings indicate increased spermatogenesis in HF bulls compared to other two breeds.

The major cell types in seminiferous tubule include spermatogenic cells (spermatogonia, spermatocytes, spermatids, and spermatozoa) and Sertoli cells (supporting/nursing cells). We used GATA4, a specific marker for Sertoli, peritubular, interstitial cells [15], for localization of Sertoli cells in bulls and found that that the Sertoli cell number was higher in HF, followed by Tharparkar and crossbred bulls. Earlier study from our laboratory proved that the proportion of Sertoli cells to the total spermatogenic cells was lower in bulls producing inferior quality ejaculate compared to those bulls producing superior quality semen [21]. The relationship between germ cell and Sertoli cell numbers has been studied as an indicator of the spermatogenesis efficiency in different species [22,23]. Lower percentages of Sertoli cells indicate that the cell population of seminiferous epithelium is composed primarily of germ cells. Sertoli cells are the somatic cells of the testis that are essential for testis formation and spermatogenesis. These cells facilitate the progression of germ cells to spermatozoa via direct contact and by controlling the environment milieu within the seminiferous tubules. They regulate the biochemical surroundings of the germ cells [24]. Sertoli cells provide critical factors for germ cell development either in the form of physical support, junction complexes or barriers, or there may be biochemical stimulation in the form of growth factors or nutrients [25]. Thus, it can be hypothesized that more numbers of Sertoli cells per spermatogenic cell would provide sufficient support and nourishment for successful progression of spermatogenesis and for production of good quality spermatozoa as observed in the present study also (non-problematic bulls-HF and Tharparkar- had higher proportion of Sertoli cells than the problematic bull-KF crossbred).

It has already been reported that the inherent potential of each Sertoli cell is independent of the absolute numerical size of the Sertoli cell population, those bulls with the highest spermatogenic to Sertoli cell ratio would already be functioning closer to their maximum potential, and their opportunity for increased daily spermatozoa production would be more limited. Because of their intimate physical relationship with the germ cells and the variety of supportive roles they perform, it seemed reasonable to speculate that the absolute numerical size of the Sertoli cell population might establish the upper limit for spermatozoal production in any given testis [26]. Thus, decreased number of Sertoli cells per unit spermatogenic cells may be a limiting factor for good quality ejaculates in crossbred bulls.

## Conclusions

From the findings of the present study, it may be inferred that variations exist in terms of seminiferous tubule area and its compactness, spermatogenic cells area, and concentration of Leydig cells in interstitial tissue among the three breeds studied and the proportion of Sertoli cells in relation to spermatogenic cells was significantly lower in KF bulls compared to either HF or Tharparkar bulls.

## Authors’ Contributions

AK designed the work. UKT and SC conducted experiments. MKMA, SKR, and SN helped UKT for microscopic and statistical analysis. AM, TKM, SY, and AK took part in preparing the manuscript. All authors read and approved the final manuscript.
